# Bone marrow cell derived arginase I is the major source of allergen-induced lung arginase but is not required for airway hyperresponsiveness, remodeling and lung inflammatory responses in mice

**DOI:** 10.1186/1471-2172-10-33

**Published:** 2009-06-01

**Authors:** Kathryn A Niese, Ann R Collier, Amanda R Hajek, Stephen D Cederbaum, William E O'Brien, Marsha Wills-Karp, Marc E Rothenberg, Nives Zimmermann

**Affiliations:** 1 Division of Allergy and Immunology, Cincinnati Children's Hospital Medical Center and the University of Cincinnati College of Medicine, Cincinnati, Ohio, USA; 2 Division of Genetics, Department of Pediatrics, University of California Los Angeles Medical Center, Los Angeles, California, USA; 3 Department of Molecular and Human Genetics, Baylor College of Medicine, Houston, Texas, USA; 4 Division of Immunobiology, Cincinnati Children's Hospital Medical Center and the University of Cincinnati College of Medicine, Cincinnati, Ohio, USA

## Abstract

**Background:**

Arginase is significantly upregulated in the lungs in murine models of asthma, as well as in human asthma, but its role in allergic airway inflammation has not been fully elucidated in mice.

**Results:**

In order to test the hypothesis that arginase has a role in allergic airway inflammation we generated arginase I-deficient bone marrow (BM) chimeric mice. Following transfer of arginase I-deficient BM into irradiated recipient mice, arginase I expression was not required for hematopoietic reconstitution and baseline immunity. Arginase I deficiency in bone marrow-derived cells decreased allergen-induced lung arginase by 85.8 ± 5.6%. In contrast, arginase II-deficient mice had increased lung arginase activity following allergen challenge to a similar level to wild type mice. BM-derived arginase I was not required for allergen-elicited sensitization, recruitment of inflammatory cells in the lung, and proliferation of cells. Furthermore, allergen-induced airway hyperresponsiveness and collagen deposition were similar in arginase-deficient and wild type mice. Additionally, arginase II-deficient mice respond similarly to their control wild type mice with allergen-induced inflammation, airway hyperresponsiveness, proliferation and collagen deposition.

**Conclusion:**

Bone marrow cell derived arginase I is the predominant source of allergen-induced lung arginase but is not required for allergen-induced inflammation, airway hyperresponsiveness or collagen deposition.

## Background

Asthma is a serious, chronic inflammatory disorder that is responsible for one in six pediatric emergency room visits, is the 3^rd ^leading cause of hospitalization among children and is one of the leading causes of school absenteeism. In the United States, nearly 30% of the population suffers from allergies with 5–10% inflicted with asthma. Despite intense ongoing asthma research, there is currently an epidemic of this disease in the western world and the incidence is on the rise [1,2]. The pathophysiology of asthma is characterized by eosinophil-rich inflammatory cell infiltrates, increased mucus production, airway hyperreactivity, and reversible airway obstruction [3-5]. Experimentation in the asthma field has largely focused on analysis of the cellular and molecular events induced by allergen exposure in sensitized animals (primarily mice) and humans. While these studies have provided the rationale for the development of multiple therapeutic agents that interfere with specific inflammatory pathways [6], the development of the asthma phenotype is likely to be related to the complex interplay of a large number of additional genes, and their polymorphic variants.

Accordingly, in an effort to identify new genes involved in the pathogenesis of asthma, we reported a group of genes that was induced in the lungs in two phenotypically similar models of experimental asthma triggered by independent regimes [7,8]. Among these asthma signature genes, we found overexpression of genes encoding for enzymes and transporters involved in arginine metabolism, specifically arginase I, arginase II and CAT2 [7]. We chose to focus on these genes because intracellular arginine is a regulator of diverse pathways including production of nitric oxide, polyamines, and proline; these molecules regulate critical processes associated with asthma including airway tone, cell hyperplasia and collagen deposition, respectively [9,10]. Furthermore, recent studies have shown a role for arginase in several parasitic models [11-16], commonly associated with Th2/M2 inflammation. Finally, recent studies with arginase inhibitors suggested an effect on outcomes of allergic airway inflammation in mice and guinea pigs [17-19]. However, the results of these studies were contradictory with one study suggesting a protective and the other two a detrimental role for arginase in allergen-induced inflammation and airway hyperresponsiveness. Altogether, we tested the hypothesis that arginase expression has a role in allergic airway inflammation by subjecting arginase I-deficient bone marrow chimeric mice and arginase II-deficient mice to allergen challenge-induced airway inflammation. We demonstrate that arginase I expression does not affect bone marrow reconstitution following transfer into lethally irradiated recipients and that arginase is not required for baseline immunity. We also demonstrate that BM-derived arginase I is the main source of allergen-induced lung arginase. However, our studies demonstrate that arginase is not required for allergen-induced airway inflammation, hyperresponsiveness or collagen deposition.

## Methods

### Generation of arginase I bone marrow (BM) chimeras

All animal studies were approved by the CCHMC IACUC committee. Arginase I heterozygous mice [20] were bred and pups were genotyped 7–9 days after birth. Bone marrow was collected from arginase I -/- pups and heterozygous or wild type (majority of experiments) pups (postnatal day 9–12). No difference was observed in experiments where +/- versus +/+ mice were used as control. In early experiments we transferred 1 × 10^6 ^total bone marrow cells, and in later ones 2 × 10^5 ^low density bone marrow cells were used. No difference in engraftment was observed with the two methods. Recipient mice (CD45.1 congenic mice) were irradiated [2 doses of ^137^Cs (700 and 475 rads) 3 hours apart] and bone marrow injected i.v. Engraftment was checked by CD45.1 (recipient)/CD45.2 (donor) on peripheral blood by flow cytometry (antibodies from BD Pharmingen specific for CD45.1 and CD45.2 are clones A20 and 104, respectively)) and allergen challenges started 8–14 weeks post-irradiation. In some experiment, C57Bl/6 mice were used as recipients and thus chimerism was not checked prior to the allergen challenge. However, in all experiments we verified that arginase activity was not induced in the lung of allergen-challenged arginase I BM chimeric mice (see results). As an additional control, in some experiments we used mice that were not irradiated and bone marrow transferred (non-BMT). Finally, we backcrossed arginase I-heterozygous mice for 6 generations on the BALB/c background and verified findings obtained with C57Bl/6 mice (specifically, the expression of arginase I in the lung by Northern blot analysis, airway hyperresponsiveness and BALF cellularity). Since congenic mice were not used to check engraftment with BALB/c mice, we verified lack of arginase I expression in the lung by Northern blot analysis.

### Induction of allergic airway inflammation

Arginase I BM chimeras and Arginase II-deficient mice [21] were allergen challenged using two models, as described previously [7,8,22]. Briefly, in the first model, mice were sensitized i.p. with ovalbumin (OVA, 100 μg) in alum (1 mg) and challenged intranasally (i.n.) with 50 μg OVA or saline. *Aspergillus fumigatus *antigen-associated asthma was induced by challenging mice intranasally three times a week for three weeks, as described [23-25]. In brief, mice were lightly-anesthetized with isofluorane inhalation and 100 μg (50 μl) of *Aspergillus fumigatus *extract (Bayer Pharmaceuticals, Spokane, WA) or 50 μl of normal saline alone was applied to the nasal cavity using a micropipette with the mouse held in the supine position. After instillation, mice were held upright until alert. Mice were sacrificed 18–24 hours following the last challenge.

### Arginase activity

Arginase activity was measured using the blood urea nitrogen reagent (Sigma Chemical Company, St. Louis, MO) according to established techniques [26-28].

### *In situ *hybridization of mouse lung

*In situ *hybridization was performed as described [7]. In brief, murine arginase I cDNA in plasmid pCMV-SPORT6 (Incyte Genomics, St. Louis, MO) was linearized by EcoRI or Not I digestion, and anti-sense and sense RNA probes, respectively, were generated by T7 and SP6 RNA polymerase (Riboprobe Gemini Core System II transcription kit; Promega, Madison, WI). The radiolabeled [αS^35^-UTP] probes were hybridized and washed under high-stringency conditions.

### Northern blot analysis

RNA was extracted using the Trizol reagent as per the manufacturer's instructions. The cDNA probes, generated by PCR or from commercially available vectors [Image Consortium obtained from American Tissue Culture Collection, Rockville, MD or Incyte Genomics, Palo Alto, CA], were sequence confirmed, radiolabelled with ^32^P, and hybridized using standard conditions, as described previously [7].

### Measurement of airway hyperreactivity

Allergen-induced AHR was determined as described previously [29,30]. Briefly, mice were anesthetized, intubated and ventilated at a rate of 120 breaths per minute with a constant tidal volume of air (0.2 ml), and paralyzed with decamethonium bromide (25 mg/kg). After establishing a stable airway pressure, 25 μg/kg weight of acetylcholine was injected i.v. and dynamic airway pressure (airway pressure time index [APTI] in cm H_2_O/sec) was followed for 5 minutes.

### Measurement of collagen accumulation

Collagen accumulation was determined as described previously [31]. Briefly, the upper, left lobe of lung was homogenized in 0.5 M acetic acid. Following the addition of pepsin (1 mg/10 mg tissue, Sigma), the lung homogenates were vigorously shaken overnight at 4°C. Collagen content was determined biochemically by quantifying total soluble collagen using the Sircol collagen assay kit (Biocolor Ltd, Newtownabbey, Northern Ireland) according to the manufacturer's instructions. The data are expressed as the collagen content normalized per mouse weight. In other experiments, we measured collagen accumulation by measuring the content of hydroxyproline, as previously described [32].

### Antigen-specific antibody measurement

Plasma OVA-specific IgG_1 _was measured after coating the wells with OVA (100 μg/ml). Blocking was done with 10% FBS in PBS, and all washes were performed with 0.1% Tween-20 in PBS. Plasma samples were diluted 1:1000 and then serially diluted 1:4. After 2 hours of incubation, plates were washed and HRP-conjugated anti-mouse IgG1 (1:1,500) (X56; BD PharMingen) was added. The OD was read at 450 nm within 10 minutes.

### Ki67 staining and quantification

Tissues were fixed in 10% neutral buffered formalin and paraffin embedded. Immunohistochemistry analysis was performed on deparaffinized 5 μm sections after blocking of endogenous peroxidase activity, antigen retrieval (in citrate buffer (pH6) in microwave oven for 7 min) and blocking with normal goat serum. The primary antibody [anti-Ki67 1:50 (clone B56, BD PharMingen)] was diluted in 0.1% bovine serum albumin in PBS, applied to tissue sections, and incubated overnight at 4°C. Antibody staining was detected with biotinylated anti-mouse IgG secondary antibody and Vectastain ABC, MOM Immunodetection, and DAB Substrate kits (Vector Laboratories, Inc.). Sections were counterstained with nuclear fast red. Counts represent evaluation of an average number of cells per mm^2 ^determined by an observer blinded to treatment and genotype using Metamorph software.

### Statistical analysis

The significance of differences between groups were analyzed using two-way (disease and genotype) ANOVA using Prism software. Values are reported as the mean ± standard deviation. Differences are considered significant if P < 0.05. Pairwise comparisons were performed by Student's t-test and P values are shown in the figures.

## Results

### Development of arginase I-deficient bone marrow chimeras

Arginase I-deficient mice [20] develop severe hyperammonemia and die between postnatal days 10 and 14. Since we have previously demonstrated that arginase I expression in allergic airway inflammation is located in macrophages [7], we developed bone marrow chimeras with arginase I-deficient bone marrow derived from arginase I-deficient pups. In experiments where congenic CD45.1 mice were used as recipients, we evaluated donor (CD45.2) chimerism in the peripheral blood at monthly intervals prior to commencing allergen challenges. Importantly, there was no difference in engraftment of arginase I-deficient and wild type bone marrow (for instance, in a representative experiment there were 92.14 ± 1.46 and 91.13 ± 2.64% donor-derived cells, respectively; n = 7–8 mice, P = 0.38) demonstrating that arginase I expression did not affect bone marrow reconstitution following bone marrow transfer.

### Bone marrow-derived arginase I is not required for basal immunity

Since previous studies have demonstrated that arginase transgenic mice have defective B cell maturation and impaired development of Payer's patches [33,34], we analyzed chimeric mice for basic hematological and immunological parameters. We found that mouse body weight, spleen weight, complete blood counts, engraftment of individual cell types (CD4, CD8 and B220-positive cells) were not dependent on arginase expression in BM-derived cells (data not shown). Peyer's patches were not detectable macroscopically following irradiation irrespective of arginase expression. In summary, we demonstrate that bone marrow-derived arginase I is not required for baseline immune cell development.

### Bone marrow-derived arginase I is not required for adaptive immunity

**Figure 1:**
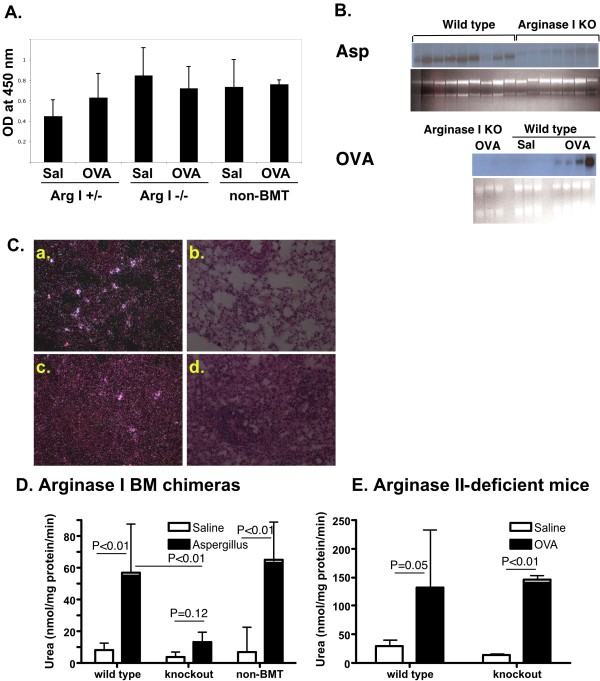
**Characterization of arginase I-deficient BM chimeric mice**. In A, OVA-specific IgG1 was measured in plasma. Representative experiment (2–4 mice per group) of 3 experiments performed is shown. In B, Northern blot analysis for arginase I in allergen-challenged (OVA- ovalbumin model and Asp- *Aspergillus fumigatus *model) wild type and arginase I-deficient BM chimeras is shown. Ethidium bromide-stained gel is shown as loading control. In C, *in situ *hybridization with arginase I anti-sense probe is shown. Panels (a) and (b) are from an allergen-challenged arginase I +/- BM chimeric mouse and (c) and (d) are from an allergen-challenged arginase I -/- BM chimeric mouse. (a) and (c) are dark-field and (b) and (d) are bright-field images of the same area. Representative micrographs of 3 mice in each group are shown. In D and E, arginase activity on lung homogenates is shown. Representative of 4 experiments (2 with OVA and 2 with Asp) for arginase I chimeras, and 3 for arginase II-deficient mice are shown. Data are from 2–6 mice per group in the individual experiment with 7–15 total mice per group. For both arginase I chimeras and arginase II-deficient mice, two-way ANOVA demonstrated a statistically significant (P < 0.01) effect of treatment (allergen compared to saline), with no statistically significant effect of interaction (P > 0.05). The P value for the effect of genotype was P = 0.055 in arginase I BM chimeras and P = 0.98 in arginase II-deficient mice.

7–14 weeks post-irradiation, mice were challenged with allergen. We used two models of allergic airway inflammation, as we have shown arginase I and II are increased in both models and are part of the "asthma signature genome" [7,8]. Importantly, while these two independent models use different antigens and routes of sensitization, the phenotype in mice is similar [7,8]. In the first model, mice are sensitized intraperitoneally (i.p.) with OVA and adjuvant alum and then challenged intranasally with OVA or saline for control. In the second model, mice were sensitized and challenged mucosally (by the intranasal route) with Aspergillus fumigatus extract. First, we verified that sensitization is not affected by arginase I deficiency. We measured OVA-specific IgG1 in OVA-sensitized mice and determined that deficiency of arginase I in bone marrow-derived cells did not affect systemic sensitization (Figure 1A), which is consistent with our finding that the hematological and immunological parameters are grossly normal in these mice. Similarly, OVA-specific IgE was not affected by arginase I expression in bone marrow derived cells (data not shown).

### Bone marrow arginase I is the main source of allergen-induced lung arginase

We hypothesized that bone marrow-derived cells are required for arginase expression in the lung of allergen-challenged mice. As seen in Figure 1B, Northern blot analysis for arginase I indeed shows decreased expression of arginase I in arginase I chimeric mice (wild type mice transplanted with arginase I-deficient bone marrow). Similarly, *in situ *hybridization of allergen-challenged arginase I-deficient BM chimeric mice demonstrated a significant decrease in the number of arginase I-expressing cells compared to mice that received arginase I +/- bone marrow cells. (Figure 1C). In order to verify that the arginase I BM chimeras indeed are deficient in lung arginase, we measured arginase activity in allergen-challenged mice. As shown in Figure 1D, Aspergillus-challenged non-BMT mice and arginase +/+ BM chimeras induce arginase activity in the lung. However, arginase I-deficient BM chimeras did not show increased arginase activity with allergen challenge. Similar results were seen in the OVA model (data not shown). We have shown that both arginase I and arginase II expression are increased in allergen challenged mice [7]. In order to test the hypothesis that arginase I is the main contributor to the observed increase in arginase activity in allergen-challenged mice, we measured arginase activity in allergen-challenged arginase II-deficient mice. As seen in Figure 1E, arginase activity was increased in the lungs of arginase II-deficient mice to a level similar to wild type mice. In summary, these data demonstrate that arginase I is primarily induced in bone marrow-derived cells and that arginase I expression predominantly contributes to allergen-elicited arginase activity in the allergic lung.

### Disease outcomes in allergic airway inflammation

**Figure 2:**
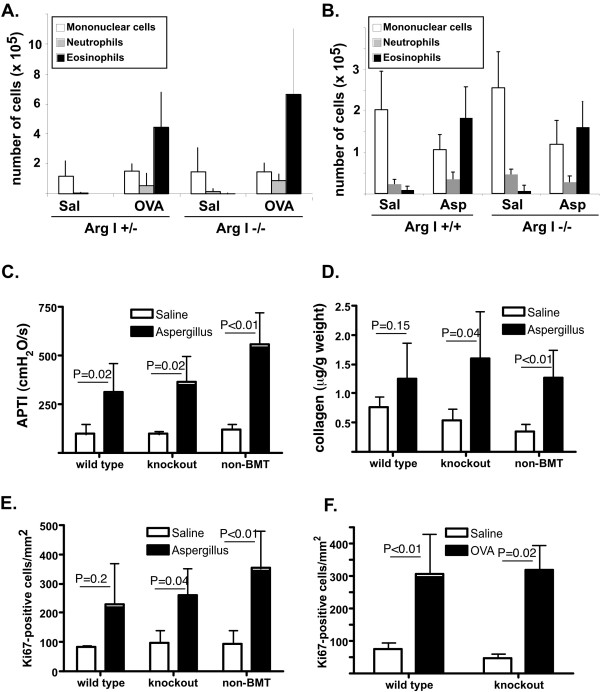
**Disease outcomes in arginase I-deficient BM chimeric mice**. In A and B, cell composition of the BALF is shown from OVA and Aspergillus model experiments. Data are from 2–4 (OVA model) and 3–11 (Asp model) mice per group. In C, airway hyperresponsiveness is shown. Data are from 3–6 mice per group. In D, collagen content of the lung is shown. Data are from 4–6 mice per group. In E and F, quantification of Ki67-positive cells by immunohistochemistry in Asp and OVA model respectively, is shown. Data are from 2–6 mice/group. In C-F, two-way ANOVA demonstrated a statistically significant (P < 0.001) effect of treatment (allergen compared to saline), with no statistically significant effect of genotype or interaction (P > 0.05). In each panel, a representative experiment out of three performed is shown.

We were first interested if arginase I has a role in inflammation and cell recruitment in allergic airway inflammation. Cells in the BALF were differentiated based on morphology and we demonstrate that arginase I BM chimeras had similar allergen-elicited recruitment of inflammatory cells (Figure 2A–B). Similar results were obtained in arginase I-deficient BM chimeras on the BALB/c background with OVA and Aspergillus-induced allergic inflammation. Furthermore, we assessed the expression of cytokines and chemokines known to be induced by allergen challenge. Northern blot analysis demonstrated induction of eotaxin-2, TARC, 15-lypoxygenase and small proline rich protein 2 (SPRR2) by OVA irrespective of arginase I expression in the lungs (data not shown). Furthermore, the protein level of eotaxin-1, MIG, RANTES, IL-4, IL-5 and leukemia inhibitory factor (LIF) were induced by Aspergillus in the lungs of mice irrespective of arginase I (data not shown). These data demonstrate that arginase I is not required for the development of inflammation in allergic lung inflammation.

Arginase can compete with NOS for substrate, arginine, and can thus lead to decreased production of NO. As NO has been shown to regulate airway tone, we tested the hypothesis that arginase I has a role in allergen-induced airway hyperreactivity (AHR). For these studies, we used only the Aspergillus model which consistently induced AHR in allergen-challenged C57Bl/6 mice. As seen in Figure 2C, Aspergillus-challenged non-BMT and arginase I +/+ BM chimeras demonstrate increased responsiveness to acetylcholine, compared to their respective saline-challenged controls. Interestingly, even in the absence of arginase I activity in the lung, mice displayed AHR. Similar results were obtained with arginase I-deficient BM chimeras on the BALB/c background. These data demonstrate that arginase I is not required for allergen-induced AHR.

Arginase metabolizes arginine to urea and ornithine which can further be metabolized by ornithine aminotransferase to proline, an amino acid that is often the rate-limiting substrate for collagen synthesis [35-37]. Thus, we tested the hypothesis that arginase I has a role in allergen-induced collagen deposition. For these studies, we used only the Aspergillus model which demonstrates significant allergen-induced collagen deposition. As seen in Figure 2D, we observed increased collagen deposition in non-BMT mice, as well as in arginase I-deficient and sufficient BM chimeras. On average, there was a 39.2 ± 5% and 58.8 ± 9% allergen-induced increase in wild type and arginase I-chimeric mice, respectively (P = 0.47, n = 10–16 mice per group). These data demonstrate that arginase I is not required for allergen-induced collagen accumulation.

Ornithine, the metabolite of arginase activity, can serve as substrate for ornithine decarboxylase, leading to production of polyamines, which plan an important role in proliferation of cells. Thus, we tested the hypothesis that arginase I has a role in allergen-induced cell proliferation. As seen in Figure 2E–F, we observed increased numbers of Ki67-positive cells in allergen-challenged mice. These levels were comparable irrespective of arginase expression. Thus, these data demonstrate that arginase I is not required for allergen-induced proliferation.

### Arginase II- deficient mice

**Figure 3:**
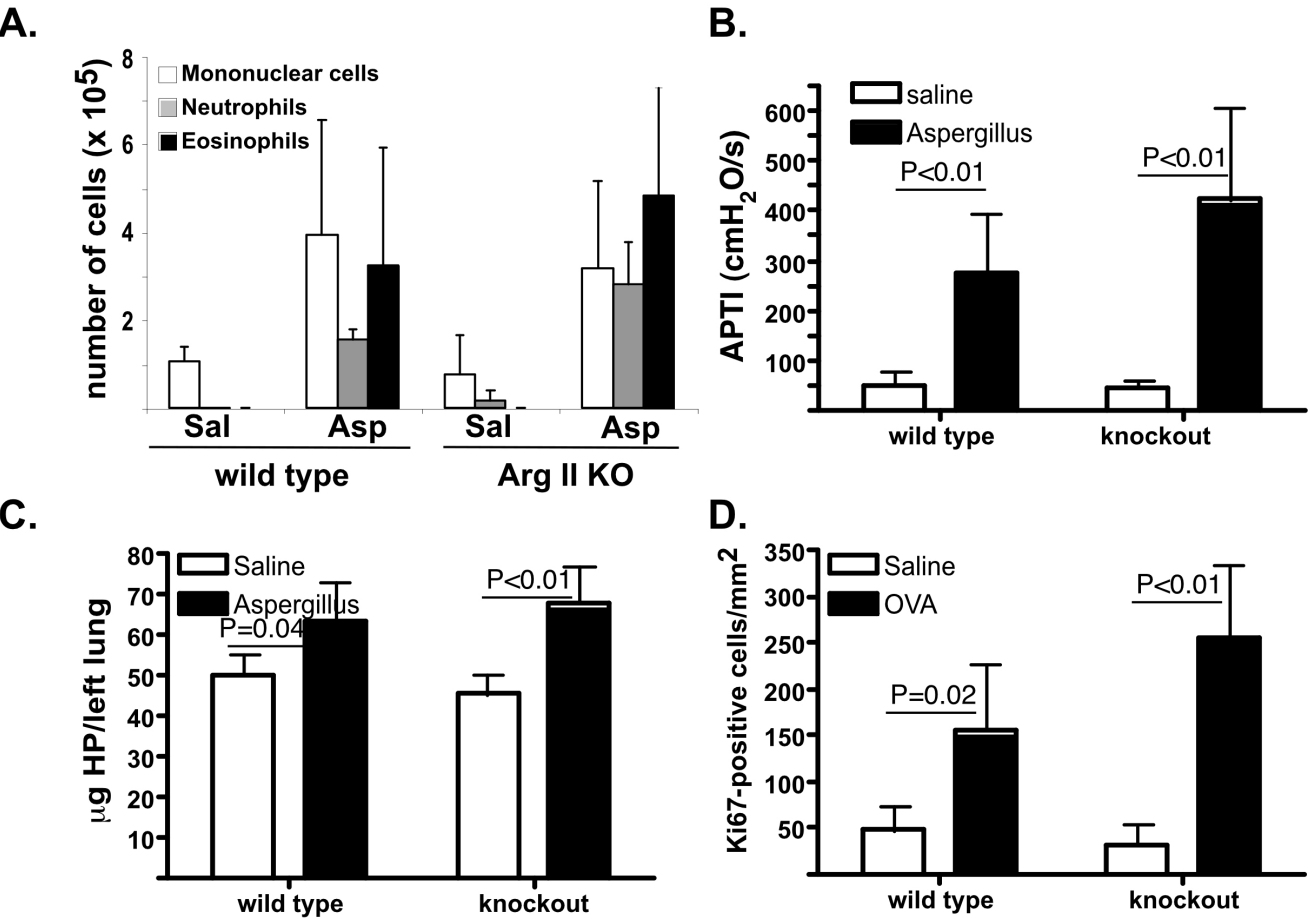
**Disease outcomes in arginase II-deficient mice**. In A, cell composition of the BALF is shown. Data are from 3–4 mice per group. Representative experiment of 5 (2 with Aspergillus and 3 with OVA model) is shown. In B, airway hyperresponsiveness is shown. Data are from 4–6 mice per group. In C, collagen content of the lung is shown. Data are mean ± SD of 4–6 mice per group. In D, quantification of Ki67-positive cells by immunohistochemistry is shown. Data are from 2–5 mice/group. In B-D, two-way ANOVA demonstrated a statistically significant (P < 0.001) effect of treatment (allergen compared to saline), with no statistically significant effect of genotype or interaction (P > 0.05). In B-D, a representative experiment of two performed is shown.

It remained possible that arginase II is the critical arginase isoform in outcomes of allergic lung inflammation. The subcellular localization and presumed function of the two isoforms is different with arginase II expressed in the mitochondria (while arginase I is cytoplasmic) and predominantly implicated in biosynthetic pathways (while arginase I is predominantly thought to be involved in the urea cycle). Thus, we allergen challenged arginase II-deficient mice [21] and analyzed for outcomes of allergic airway inflammation. As seen in Figure 3A, arginase II-deficient mice had allergen-induced BALF cell accumulation comparable to arginase II-wild type mice. Similarly, the arginase II-deficient mice had levels of allergen-induced airway hyperreactivity similar to wild type mice (Figure 3B). Furthermore, collagen deposition was induced in arginase II-deficient mice to levels comparable to those of arginase II-wild type mice (Figure 3C). Finally, proliferation of cells was not affected by the presence of arginase II (Figure 3D). Importantly, arginase activity in the lung was induced to similar levels in arginase II-deficient and wild type mice (Figure 1E). Thus, these data demonstrate that arginase II is not required for allergen-induced airway inflammation, AHR, collagen deposition and proliferation.

## Discussion

The alternative pathway of macrophage activation, via IL-4, has been recently appreciated in many Th2-associated responses, such as parasitic infections and allergic inflammation. However, the role of these cells and their effectors in the broad spectrum of Th2-associated pathological processes is poorly understood. We investigated the role of the enzyme arginase, which is a marker of alternatively activated macrophages and highly upregulated in allergic and parasitic infection. While the role of arginase in the urea cycle is well understood, its role in inflammation is less clear. Based on known downstream mediators, arginase has been speculated to play a role in NO production and subsequent inflammation and regulation of airway tone, as well as proliferation and collagen synthesis via ornithine production. We used arginase I-deficient bone marrow chimeric mice in models of allergic airway inflammation. Use of arginase I BM chimeric mice revealed several novel findings. First, we demonstrate that arginase I expression does not affect bone marrow reconstitution following transfer into lethally irradiated recipients and that arginase is not required for baseline immunity. Second, we demonstrate that BM-derived arginase I is the main source of allergen-induced lung arginase. Third, we found that lung arginase is dispensable for regulation of inflammation, airway tone, fibrosis and cell proliferation during allergic airway inflammation.

Studies using arginase transgenic mice have demonstrated that overexpression of arginase (specifically in gut epithelial cells) leads to interrupted development of B cells and impaired Peyer's patch architecture [33,34]. Mechanistic analysis suggested that the hypoargininemia was responsible for the phenotype as arginine supplementation reversed the changes. Thus, it was important to assure that extrahepatic arginase deficiency does not perturb bone marrow engraftment and baseline immune parameters. We show here that genetic deficiency of arginase in bone marrow-derived cells does not affect BM engraftment, baseline and adaptive immunity. Thus, we speculate that excessive and/or systemic expression of arginase, such as in transgenic mice overexpressing arginase may affect the immune system via decreasing systemic arginine levels; however, lack of extrahepatic arginase does not appear to grossly affect the immune system.

Previous studies have suggested that macrophages are the main source of arginase in the allergen challenged mouse lung. However, other cell types, including respiratory epithelial cells have been implicated as potential sources of arginase [7,38-40]. Furthermore, we and others have shown that both arginase I and II expression is increased in the lungs following allergen challenge [7]. Thus, it remained possible that arginase II will contribute to lung arginase activity in allergen challenged arginase I BM chimeric mice. Our studies measuring arginase activity in both arginase I BM chimeric and arginase II-deficient mice demonstrated that arginase I, expressed in bone marrow cells, is the main contributor to arginase activity in allergic lung. These data support the notion that bone marrow-derived cells, such as infiltrating inflammatory macrophages, are the main source of the arginase I enzyme, and arginase activity in the allergic lung.

We originally hypothesized that arginase induction has a role in the pathophysiology of allergic airway inflammation. This was based on several findings. First, we and others have shown that arginase expression and function is increased in the lungs of allergen challenged mice and in the lungs and serum of humans with asthma [7,41]. However, these studies did not address the specific role of arginase in pathophysiology of the disease. Second, downstream pathways of arginase have been implicated in the regulation of inflammation, collagen deposition, proliferation and airway hyperresponsiveness [35,42,43]; these are all hallmark outcomes in asthma. Specifically, Meurs et al have elegantly demonstrated a role for arginase in contractility of allergen-challenged guinea pig tracheas *ex vivo*. They have also demonstrated that the mechanism for this effect includes arginase-mediated decrease of NO production [44,45]. These results, obtained in guinea pigs and in reductionist *ex vivo *approaches, differ from our data that were obtained in the complex *in vivo *environment of allergen challenged mice. We speculate that the difference lies either in the model system (guinea pigs versus mice) or that parallel pathways are invoked *in vivo *during allergen challenge, thus making the role of arginase redundant. Third, mouse models of parasite infestation, including Schistosome mansoni, Heligmosomoides polygyrus, Leishmania sp, Toxoplasma gondii and Nipostrongylus brasiliensis [11-16] demonstrated a role for arginase expression in host defense. Interestingly, in at least one of those publications the role of arginase was confined to models of infection by intracellular pathogens; however, arginase did not play a role in a systemic disease despite induction of arginase in both models [16]. Finally, we have shown a role for arginase in IL-13 induced airway hyperresponsiveness [46] and others have recently suggested a role for arginase in mouse and guinea pig asthma models using arginase inhibitors [17-19]. Specifically, we have shown that delivery of arginase I siRNA to mice that receive IL-13 i.t. leads to decreased arginase I expression and airway responsiveness. It is important to note that this effect was observed at early time points (12–48 hours following single i.t. installation of IL-13). Thus, we speculate that arginase may play a role in airway responsiveness to a single cytokine at early time points when collateral pathways have not been able to develop; in contrast, in allergen challenge multiple pathways are activated that may contribute to airway responsiveness thus making arginase redundant. The study by Maarsingh et al [18], demonstrated that inhalation of ABH, an inhibitor of arginase, protects against allergen-induced airway obstruction, hyperresponsiveness and inflammation. We speculate that our results differ from the ones in this study either because of the different species used (mice versus guinea pigs) or because of the different approach (inhibitors versus genetic ablation of arginase). It is possible that the inhibitor has non-specific off-target effects that may be responsible for the observed effects. It is important to note that arginase plays an important role in the urea cycle in the liver; mutations in arginase leads to severe metabolic consequences in humans and mice, including hyperargininemia, hyperammonemia and premature death [20,47]. In an effort to confirm our results, we used an inhibitor of arginase, BEC, and found that it leads to systemic effects, including hyperargininemia (data not shown). Since plasma arginine levels can have profound effects on the immune system [33,34,48-54], we elected not to pursue studies with inhibitors as we believed we could not distinguish the effects of arginase inhibition locally in the lungs from indirect effects from arginase inhibition in the liver. In contrast to the study by Maarsingh et al where arginase inhibitor decreased inflammation and airway hyperresponsiveness, Ckless et al [17], found that inhibitor of arginase led to increased inflammation and airway hyperresponsiveness in mouse models of allergic inflammation. Together, these data caution against use of arginase inhibitors in allergic airway inflammation until the effects (specific and off-target) and mechanisms are fully elucidated.

In summary, our data suggest divergent role for arginase I in allergic inflammation compared to parasitic responses. Since arginase is a prominent product of alternatively activated macrophages, which are induced by IL-4 in both allergic and parasitic responses, our data suggests alternatively activated macrophages evolved to combat parasitic infections and are either bystanders in allergic inflammation or have developed other effector molecules for allergic Th2-associated responses.

## Conclusion

Bone marrow cell derived arginase I is the predominant source of allergen-induced lung arginase but is not required for allergen-induced inflammation, airway hyperresponsiveness or collagen deposition.

## Abbreviations

AHR: airway hyperresponsiveness; BALF: bronchoalveolar lavage fluid; BM: bone marrow; BMT: bone marrow transfer; OVA: ovalbumin.

## Authors' contributions

KAN, ARC, ARH and NZ performed the research. SDC and WEO provided critical reagents (arginase I and II-deficient mice, respectively). MWK provided the AHR measurements. MER participated in the conception and design of the study, and helped draft the manuscript. NZ participated in the conception, design and coordination of the study, analyzed the data and drafted the manuscript. All authors read and approved the final manuscript.

## Authors' information

ARC is currently at Oak Brook Allergists in Oak Brook, Illinois. ARH is currently enrolled in the School of Veterinary Medicine, University of Illinois at Champaign-Urbana.
